# ANTIDEPRESSANT USE AND MORTALITY RISK IN OLDER ADULTS WITH HISTORY OF STROKE

**DOI:** 10.1093/geroni/igz038.999

**Published:** 2019-11-08

**Authors:** Madison B Lenox, Victor Tran, Grace Tworek, Andrew Solow, Diana Hincapie

**Affiliations:** Nova Southeastern University, Fort Lauderdale, Florida, United States

## Abstract

The American Stroke Association reports stroke as the fourth leading cause of death in the United States, with 66% of hospitalized cases being older adults. Recovery from stroke is a public health issue, as post-stroke depression (PSD) is a significant concern. Approximately 20-23% of stroke survivors identified co-occurring diagnoses, which are associated with physical, functional, and cognitive limitations and increased mortality risk. Antidepressant use has exhibited its efficacy in treating PSD. This study explores the association between antidepressant use and mortality risk in older adults with history of stroke. Older adults aged 65 and older (N=3631, 55.4% female, 72.6% Caucasian, Mage=79.64 years, SDage=7.29 years, MEd=14.55 years, SDEd=8.269 years) with history of stroke were selected from the National Alzheimer’s Coordinating Center database to explore the association between antidepressant use and mortality. A chi-squared test of independence was calculated comparing antidepressant use and mortality rates. A significant association was found (χ2 (1) = 15.933, p < .001) between current antidepressant use and mortality. Findings suggest antidepressant use is associated with lower mortality rates in subjects with a history of stroke. Implications include highlighting the role psychologists play in the early identification of PSD and early antidepressant intervention post-stroke to increase life longevity. Although findings only infer association, they demonstrate evidence for the link between PSD, antidepressant use, and lower mortality rates. Future directions include exploring other forms of depression treatment and mechanisms of antidepressant use. Limitations include examining potential moderators (e.g., gender, SES, type of stroke), and substance use within this population.

